# Supplementary material for: Are carbon emissions trading and green financial instruments synergistic? -Comprehensive quantitative research based on content analysis
Source: PLoS One. 2024 Mar 7;19(3):e0298601. doi: 10.1371/journal.pone.0298601 (PMC10919601; doi:10.1371/journal.pone.0298601)
Supplement: S1 Data — (ZIP) [file pone.0298601.s001.zip › Data/地级市Z_F工作报告.zip!/╣π╬≈/─╧─■╩╨/└┤╫╘╣1⁄2╓┌║┼í╛┬φ┐╦╩2╛▌═°í┐_2016.docx]

各位代表：

　　现在，我代表市第十三届人民政府，向大会作政府工作报告，请予审议，并请各位政协委员和其他列席人员提出意见。

　　一、过去五年工作回顾

　　市第十三届人民代表大会第一次会议以来，我们走过了极不平凡的发展历程。在党中央、国务院和自治区党委、政府以及市委的正确领导下，我们开拓创新，砥砺奋进，全力以赴稳增长、促改革、调结构、惠民生、防风险，完成了市十三届人大历次会议确定的主要任务，经济综合实力大幅跃升，对外影响力显著增强，城乡面貌日新月异，人民群众获得感不断增强，为我市全面建成小康社会奠定了坚实基础。南宁正沿着科学发展的道路，昂首阔步，奋勇前进！

　　——五年来，我们围绕稳增长目标，坚持转方式调结构，经济发展提质增效，综合实力明显提升。

　　经济实现跨越式发展。我们认真贯彻落实中央、自治区一系列稳增长决策部署，先后出台我市稳增长“40条”“19条”措施，经济发展稳中有进，主要经济指标年均增速高于全国、全区平均水平。地区生产总值连续跨越2000亿元、3000亿元两个台阶，2015年达3410.09亿元，总量是2010年的1.89倍，占全区的比重提高了1.48个百分点；2015年人均GDP突破7000美元大关。固定资产投资、全部工业总产值连续突破2000亿元、3000亿元大关，2015年分别完成3366.89亿元、3323.82亿元，均比2010年翻一番，总量占全区比重分别比2010年提高2.11、1.38个百分点。2015年，财政收入突破500亿元、达572.48亿元，总量是2010年的1.9倍、占全区比重为24.54%；社会消费品零售总额完成1786.68亿元，是2010年的1.97倍，总量占全区比重为28.2%。

　　工业转型升级加快。深入实施“工业强市”战略，出台减轻企业负担、降低企业成本的若干意见，为企业减负增效。工业总产值、工业增加值、亿元企业数、园区工业总产值、规模以上工业利税、规模以上工业利润、工业投资、技改投资8个指标总量实现翻一番，2015年工业对经济增长的贡献率比2010年提高11.57个百分点。2015年工业利税总额达350.12亿元，跃居全区首位。新增产值百亿元工业产业13个、百亿元企业2家、亿元企业336家。三大国家级开发区工业总产值占全市比重由2010年的34.7%提高到2015年的56.9%，高新区成为千亿元产值开发区。规模以上万元工业增加值能耗年均下降6.3%。南南铝加工公司研发的航空铝、高铁铝产品达到国际先进水平。先进装备制造业取得历史性突破，实现南宁地铁南宁造、南宁公交南宁造。

　　现代服务业快速发展。实施金融业、现代物流业和信息服务业三年行动计划，出台加快服务业发展方案，2015年第三产业增加值1694.08亿元，是2010年的1.87倍，对经济增长的贡献率达54.95%，总量占全区比重为25.89%。新增主板上市企业6家、新三板挂牌企业14家，驻邕外资银行达4家，2015年金融业增加值占全市服务业比重达21.2%、比2010年提高6.5个百分点。拥有A级以上物流企业11家，初步建成中国—东盟国际物流基地和江南、安吉、金桥三大物流园区，货物运输总量年均增长10.5%。获批中国服务外包示范城市，形成以南宁软件园和中盟科技园为核心的产业集群。国家电子商务示范市建设扎实推进，2015年重点企业电子商务交易额突破1800亿元。成为首批25个国家信息消费示范市之一，软件和信息服务业主营业务收入占全区的80%以上。建成华润万象城、青秀万达广场等一批城市综合体，朝阳、埌东—凤岭等商业中心拓展完善，新增限额以上商贸企业640家、零售总额年均增长16.7%。会展业加快发展，举办超万平方米以上的展览120多场，会展中心改扩建工程一期完工。累计接待旅游总人数3.52亿人次、旅游总消费2983.49亿元，荣获“2014中国最佳生态旅游度假目的地”“中国养生休闲之都”称号，青秀山荣膺国家5A级旅游景区。

　　现代农业发展迅速。严格基本农田保护，持续开展高标准粮食生产基地建设，粮食产量实现“五连增”。新增“双高”糖料蔗示范基地149个、“菜篮子”基地123个、农产品标准化示范基地80个，创建畜禽标准化示范场49个，改建水产基地69个，实施林下经济示范项目74个。积极实施农业品牌化战略，建成1个国家地理标志保护示范区，南宁香蕉等10个产品获国家地理标志产品（农产品）登记保护，新增“三品一标”认证农产品58个。创新“产村互动、农旅融合”发展模式，成功创建2个国家现代农业示范区，5个自治区级、26个市级现代特色农业示范区和25个市级生态综合示范村。大力培育新型农业经营主体，新增市级以上农业重点龙头企业91家、农民专业合作社1832家、家庭农场453家。农业综合机械化水平从2010年的36.5%提升至2015年的50.2%。农业科技进步贡献率达58%。累计完成水利设施建设投资90.65亿元，病险水库除险加固完成514座，新增、改善或恢复有效和节水灌溉面积209.35千公顷。

　　——五年来，我们突出产业招商，坚持招大商引强企，投资结构不断优化，发展后劲明显增强。

　　招大引强成效明显。共引进百威英博、普洛斯等世界500强企业18家，研祥、海王等国内500强及行业100强企业38家。累计实际到位区外境内资金和实际利用外资分别为3932.53亿元、32.07亿美元，分别约占全区比重的13.1%、16.21%。外来投资对我市经济增长的贡献率达31%。

　　投资环境不断改善。深化行政审批制度改革，三大国家级开发区成立行政审批局，五象新区审批办提速提效，市级行政审批局实质性运转步伐加快。在全区率先公布54个部门单位权责清单，精简权力事项64%。建立市领导联系重大项目服务重点企业长效机制。在全区率先推进商事制度改革，“先照后证”“三证合一、一照一码”“双随机、一公开”等全面推行。全市123个乡镇（街道）、1693个村（社区）设立政务服务中心，企业对我市投资环境整体满意度连续四年排全区首位，市场主体年均增长46%。多渠道强化土地要素保障，新增建设用地指标1.64万公顷。探索区域连片土地统征统储和集中安置，征地拆迁和谐推进，五年征收集体土地2.3万公顷、拆迁1996万平方米。

　　投资结构不断优化。重点加大产业、民生、基础设施三大类投资，累计列入区、市层面统筹推进重大项目1483项、完成投资2856亿元。累计完成工业投资3777.92亿元、年均增长22.84%，技改投资完成3702.55亿元、年均增长20.6%，社会事业投资完成759.7亿元、年均增长19.2%，城市基础设施投资完成3084.09亿元、年均增长12.92%，房地产开发投资完成2379.22亿元、年均增长15.74%。鼓励民间资本进入市政、交通、金融、卫生、教育等领域，2015年民间投资完成2128.07亿元，占固定资产投资比重达63.21%。创新投融资方式，成立PPP管理中心，建立动态管理项目库，那考河流域治理等5个PPP项目成功签约社会资本方。发挥财政资金杠杆作用和“乘数效应”，设立了规模为100亿元的产业发展基金和830亿元的城市发展基金。

　　——五年来，我们统筹城乡发展，坚持走新型城镇化道路，城市功能不断完善，城市建设提升新水平。

　　坚持城市规划引领。获批并实施《南宁市城市总体规划（2011—2020）》，实现中心城区控规全覆盖。2015年底建成区面积287.4平方公里，市区常住人口365万，市域常住人口城镇化率59.31%、比2010年提高6.67个百分点。编制完成《南宁市空间发展战略规划》和“海绵城市”总体规划、五象新区总部基地城市设计、教育园区及三塘—五塘片区规划等40多项重点规划和一批专项规划。推进“多规合一”，初步形成GIS“一张图”。加强城市重点区域、公共空间等设计管控，扎实推进城乡风貌改造，推广农村住宅推荐户型。全面推进县城总体规划编制，完成市及六县土地利用总体规划、102个乡镇土地利用总体规划编制。

　　五象新区提速发展。按照“高水平规划、高质量建设、高速度推进”的要求建设五象新区，累计完成投资1060.79亿元，开工项目495个、竣工项目152个。加快产城融合，共引进产业项目167个、30家世界和国内500强企业、10家金融保险企业、15家境外上市公司，南宁中车轨道交通装备基地、源正新能源汽车、福建吴钢、万达茂等一批重大项目开工或投产；通车道路85条，“三纵三横”主干路网基本完善；邻家广场、公交场站等一批公共服务设施加快推进，南宁三中五象校区等一批知名学校落成，广西国际壮医医院等一批综合医院开工建设，核心区电力、给排水、污水处理、垃圾中转站、公厕等市政配套设施同步推进。绿色建筑比例达93%，五象湖公园、总部休闲公园建成开放。总部基地金融街、五象湖周边、蟠龙至龙岗片区沿江地带建设初具规模，新区建成区面积35平方公里、净增15倍。新区已成为南宁开放发展的亮丽名片。

　　城市功能不断完善。区域性国际综合交通枢纽初步形成。南广、南钦、南昆（南宁至百色段）、柳南高铁建成，火车东站、西站启用，南宁迈入“高铁时代”。吴圩机场第二航站楼运营，通航城市87个，旅客年吞吐量突破1000万人次，基本形成覆盖东盟和国内主要城市的“东盟通”和“省会通”航线网络格局。建成南宁港牛湾、六景港区，老口航运枢纽工程竣工，邕宁水利枢纽加快推进。来宾至马山等高速公路和一批农村公路建成通车，实现“县县通高速”和“村村通水泥路”。城市路网不断完善，良庆大桥、五象—平乐立交等25座跨江桥梁、城市立交桥建成使用。青环路、长堽路等一批城市道路建成通车。完成民族大道改造提升工程，实现线缆全部入地。完成机场路扩建，建成机场第二高速，南宁东等高速公路收费站外迁运营。完成406条主次干道“白改黑”，打通枫林路等10条“断头路”。轨道交通1号线东段试运营，成为全国首个开通地铁的自治区首府城市，南宁迈入“地铁时代”。新建公交场站17个、公共自行车租赁站点696个，新增新能源与清洁能源公交车2000多辆、公共自行车2万多辆。全国地下综合管廊试点城市建设加快，长虹路示范段建成使用。棚户区改造累计开工4.2万户，“老南宁·三街两巷”等71个旧城改造项目完成投资244.1亿元，朝阳、东沟岭等片区旧貌换新颜。

　　县（区）域经济加快发展。落实《南宁市主体功能区规划》，开展县（区）差异化考核。武鸣顺利实现撤县设区。横县获评“世界茉莉花和茉莉花茶生产中心”，五年规上工业总产值总量均居各县（区）首位。宾阳荣获“广西科学发展十佳县”，黎塘工业园区产值突破百亿元。上林、马山荣获“中国长寿之乡”，特色旅游快速发展。隆安定江村、定典屯分别荣获“第四批全国一村一品示范村（镇）”“中国特色村”。兴宁获评“中国商埠文化之乡”，五年社会消费品零售总额均居各县（区）首位。江南千亿元电子信息园等专业园区建设加快，扬美古镇入选全国“最美古村落”。青秀跻身全国科学发展百强区，财政收入突破百亿元大关，培育亿元税收楼宇8栋。西乡塘棚户区改造成效明显，“美丽南方”成为全国休闲农业等乡村旅游示范点，忠良村获评“中国最美休闲乡村”。邕宁五年固定资产投资、农民人均纯收入和良庆五年财政收入增速均居各县（区）首位。

　　——五年来，我们以举办重大活动为契机，坚持生态环境优先，提高城市文明程度，宜居城市魅力彰显。

　　城市影响力不断提升。圆满服务第8—13届中国—东盟博览会和中国—东盟商务与投资峰会，东博会成为三大国家一类展会之一，南宁国际会展中心被评为“中国十大最佳会展中心”。成功承办第四十五届世界体操锦标赛、中越青年大联欢、南宁国际半程马拉松赛等20多项国际赛事活动。各项赛事活动运转顺畅有序，服务保障周到细致，筹办工作廉洁高效，展示了南宁生态宜居、文明和谐的新形象。

　　建绿治水成效明显。深入实施“绿满南宁”“绿满邕江”及“中国绿城”提升工程三年行动计划，打造机场高速路、民族大道、五象大道等一批城市绿化美化彩化精品线路。2015年末，建成区绿地率、绿化覆盖率分别达36.98%、42.95%，全市森林覆盖率47.66%。荣获“国家森林城市”和首批“国家生态园林城市”，成功申办第十二届中国国际园林博览会。邕江综合治理扎实推进，精品示范段基本建成，石埠堤12.5公里防洪堤全线贯通。18条城市内河和湖泊综合整治成效显著，民歌湖—竹排江—南湖水系和相思湖—可心江—明月湖水系实现连通，“水畅、湖清、岸绿、景美”的景观效果初显。国家海绵城市试点建设扎实开展，石门森林公园、南湖环湖路海绵化改造等68项重点项目顺利完工。

　　环境保护从严有力。严格落实环境保护“党政同责、一岗双责”，四项总量减排指标全面完成。国家节能减排财政政策综合示范市建设深入推进。实施环境倒逼产业转型升级攻坚战，全面关停搬迁市区重污染企业，宾阳县制革产业整体退出。实施截污治污三年攻坚计划，建成污水管网679公里，完成190个生活污水直排口和建成区5.8公里黑臭水体整治，三塘、五象2座污水处理厂及黎塘镇等13个重点镇的污水处理设施试运行，实施埌东污水处理厂中水回用工程、实现城市核心区八大污水流域管网互通，2015年城市污水处理率达97%、比2010年提高7个百分点。餐厨废弃物资源化利用水平国内领先，城南生活垃圾填埋场正式封场，平里静脉产业园基本建成，生活垃圾焚烧发电厂发电，城市生活垃圾无害化处理率保持100%。大力推进“美丽南宁”乡村建设，建成国家级生态乡镇4个、生态村屯1个，自治区级生态乡镇12个、生态村屯226个。建成集中式、分散式污水处理设施741套，乡镇级垃圾中转站17座，农村环境明显改善。邕江集中式饮用水源地水质达标率100%，主要江河水环境功能区达标率100%，完成449个千人以上农村饮用水水源划定。环境空气质量保持在全国省会城市前列，特别是扬尘污染专项治理成效明显，“南宁蓝”成为常态。

　　城市更加文明有序。深入推进社会主义核心价值体系建设，扎实开展文明城市创建“九项重点工作”，实现“全国文明城市”三连冠。创新城市治理，深入开展“美丽南宁·整洁畅通有序大行动”，车辆平均通行能力提高20%以上，路灯亮灯率达99%，市政设施完好率达86%以上，新增路面机动车、非机动车停车位4.6万多个，实施路内停车泊位限时免费停放。规范整治“城中村”54个、农贸市场147个、无物业管理小区778个，实现“国家卫生城市”二连冠。大力整治“两违”，累计清理拆除违法建筑1938.88万平方米、违法占地2979.73万平方米。“礼让斑马线”活动广泛开展，公交、出租行业礼让率达99.7%。无偿献血逐渐成为市民的自觉行动，荣获“全国无偿献血先进城市”。数字化城市综合管理指挥系统、内河防汛信息指挥系统和建筑垃圾运输车辆监管平台建成使用。开展工程质量治理两年行动，荣获“全国城市品牌建设奖”。

　　——五年来，我们突出打造“南宁渠道”，坚持全方位开放合作，积极发展外向型经济，城市国际影响力显著提升。

　　开放合作不断拓展。积极参与“一带一路”、中国—东盟自贸区升级版建设，在南宁设立总领事馆的东盟国家达6个，国际友城增至20个。30多个中国与东盟各领域交流合作的机制在邕建立，中国—东盟信息港南宁核心基地建设启动，中国—东盟技术转移中心等一批平台落户南宁。深入实施“双核驱动”战略，与北钦防实现户籍、通讯、公积金购房、医疗保险等同城化；深化与泛珠三角省会城市合作，16个列入珠江—西江经济带基础设施建设大会战项目扎实推进。加快建设CEPA示范城市，与粤港澳台的交流合作进一步加强。

　　对外经贸加快发展。与全球159个国家和地区有贸易往来，累计外贸进出口总额217.62亿美元，年均增长21.54%。成立外商投资企业261家、合同外资额23.21亿美元。“走出去”企业达81家、对外投资总额20亿美元。

　　贸易便利化水平提升。实施无纸化通关和广西、广东、福建、海南四省区区域通关一体化、关检合作“三个一”通关模式，实现进出口货物在北部湾经济区自由流转。全国跨境贸易电子商务服务试点和中国—东盟（南宁）跨境电子商务产业园建设扎实推进，南宁跨境贸易电子商务综合服务平台建成运行。南宁保税物流中心升级为综合保税区，一期工程通过国家验收。

　　——五年来，我们注重体制机制创新，坚持全面深化改革，提高科技自主创新能力，发展活力不断增强。

　　改革不断深化。承担自治区20项、市本级46项重点改革和69项国家、自治区级试点任务顺利推进。市、县（区）政府职能进一步转变，机构改革全面完成。精简行政审批事项，推进相对集中行政许可权改革。实施综合行政执法体制改革。加快社会组织登记制度改革、行业协会商会与行政机关脱钩。完成36家全民所有制企业和企业化管理事业单位改革。28家国有独资及国有控股企业按行业、资源整合重组成九大集团。沿边金融综合改革扎实推进，南宁金融投资集团、金融资产交易中心、股权交易中心等金融平台运营，中国—东盟（南宁）货币指数对外发布。全面推开营业税改增值税试点。推进农村土地承包经营权确权登记颁证，开展“小块并大块”耕地整治，出台存量农村宅基地确权登记办法，创造性地解决了以“户”申请宅基地的政策障碍。完成公务用车改革。全面推进政府购买服务。建立政府全口径预算体系和跨年度预算平衡机制，深入开展预算绩效评价和预决算信息公开，市本级所有预算单位公开了部门预决算及“三公”经费。

　　创新能力增强。累计取得科技成果1.2万项，新建院士工作站15个、企业工程技术研究中心84家、龙头企业技术创新中心45家、创业孵化基地34个、科技企业孵化器15家。新增国家认可实验室9家、有效广西名牌产品93个。累计专利受理量3.9万件，2015年受理量是2010年的8.1倍。2015年每万人发明专利拥有量达4.71件。高新技术企业达234家，高技术产业增加值年均增长超过20%。荣获“全国科技进步先进市”七连冠。南宁·中关村双创示范基地揭牌运营，一批世界500强和行业龙头领军企业签约入驻，为实施创新驱动发展、推动产业转型升级提供了新载体新动力。建成广西首个“创客城”，荣获“全国创业先进城市”，大众创业、万众创新走在广西前列。

　　——五年来，我们树立以人民为中心的发展思想，坚持把民生工作摆在第一位，努力提高城乡居民收入和公共服务均等化水平，民生改善成效显著。

　　全市财政涉民生支出累计达1512.91亿元，占一般公共预算支出的72.44%，年均增长17.75%。2015年，城镇居民人均可支配收入29106元、农村居民人均纯收入9408元，分别是2010年的1.61倍、1.88倍。精准扶贫成效显著。落实责任、权力、资金、任务“四到县”，突出产业扶贫和基础设施扶贫。出台精准扶贫“1+10”系列文件，全面完成农村贫困户、贫困人口的精准识别和建档立卡工作，迅速启动扶贫“七个一批”“七大工程”，调整充实扶贫工作机构，建立乡镇扶贫工作站，落实帮扶干部4.92万名与贫困户结对开展“一帮一联”活动。累计解决236.79万人饮水安全问题，减少贫困人口59万人，为全面打赢脱贫攻坚战奠定了坚实基础。教育事业优先发展。2015年学前三年毛入园率、九年义务教育巩固率、高中阶段毛入学率分别为93.2%、96.01%、93.38%，分别比2010年提高24.13、11.54、8.38个百分点。五年新建和改扩建幼儿园763所、中小学校48所，新增多元普惠幼儿园439所。共接收进城务工人员随迁子女入学59.8万人次，约占全区的1/3。实现市辖县农村义务教育学生营养改善计划全覆盖，惠及学生年均40万人。兴宁、青秀高水平通过县域义务教育发展基本均衡县（区）国家认定。南宁学院实现“万人、千亩、本科”目标。南宁教育园区加快建设。促进社会充分就业。开通和拓展“12333”热线服务，完成全部乡镇（街道）、社区及300个重点建制村就业服务平台网络信息建设，累计城镇新增就业41.28万人，五年城镇登记失业率均控制在3.5%以内。社会保障更加健全。城乡居民基本养老保险实现全覆盖。企业退休人员基本养老金实现“十一连涨”。城乡居民大病保险制度全面实施。3次提高城乡居民最低生活保障标准，累计发放城乡低保金12.7亿元、农村五保金3.19亿元、城乡医疗救助金2.7亿元。建成养老服务中心13个，修改扩建乡镇敬老院247家、农村五保村646个、社区居家养老日间照料中心60个，民办养老机构增至48家，市第二社会福利院等一批养老项目开工建设。拓宽高龄老人津贴享受人群，提高补助发放标准。完成农村危房改造6.07万户。开工新建各类保障性住房10.3万套、基本建成8.12万套。卫生计生事业发展加快。基层医疗机构及县级公立医院综合改革扎实推进，新增医疗卫生机构171家（社会办医院26家），完成1384个村卫生室规范化建设，医疗卫生机构增加床位1.29万张、2015年比2010年增长45.67%，2015年新型农村合作医疗参合率达99.61%，疾病防控能力进一步增强。落实计划生育政策，有效遏制出生人口性别比偏高势头，人口素质不断提高。文化软实力大幅提升。建成南宁博物馆、档案馆、地方志馆及643个村级公共服务中心、1448家农家书屋，全市广播电视综合覆盖率达99.48%。成功打造《百鸟衣》《水街》等一批优秀精品剧（节）目，摘取国家级奖项134个。“百姓大舞台·想秀你就来”民歌湖周周演活动成为群众文化活动品牌。加强对外文化交流，成功举办中国—东盟（南宁）戏剧周、《春天的旋律》跨国春晚等。体育事业全面加强。人均体育场地面积1.36平方米，经常参加体育锻炼人口达46%，南宁籍运动员累计荣获国际比赛金牌78枚、银牌37枚、铜牌24枚。社会治理水平提升。平安南宁建设深入推进，将城市社区划分为6673个网格进行管理，全力推进“天网”工程建设、建成1.02万路高清摄像机，立体化社会治安防控体系初步构建，应急指挥中心建成使用，街面见警率和各类案件处置率大幅提高，人民群众安全感从2011年的73.02%提升至2015年的86.22%，实现社会管理综合治理“长安杯”三连冠。安全生产形势持续稳定向好，全市亿元GDP生产安全事故死亡率下降50.7%。乡镇（街道）全部组建食品药品监督管理所，建立完善市县乡村四级网格监管体系。落实为民办实事工程。共筹资305.5亿元保障自治区级为民办实事工程240个子项、市本级244个子项顺利实施，解决了一批群众关切的上学难、看病难等问题。入选2015中国十大幸福城市。

　　——五年来，我们强化政府自身建设，坚持依法履职，着力转变工作作风，科学发展动力更加强劲。

　　民主法制建设成效明显。坚持依法决策、科学决策、民主决策。全面推行政府法律顾问制度、行政执法责任制，强化行政权力的制约和监督，法治政府建设有序推进，《中国法治政府评估报告（2014）》综合评价各直辖市、省会城市及其他地级市共100个城市政府法治建设水平，我市位列100个城市的第8位、西部城市的第2位。法治南宁制度体系日趋完备，共提请市人大常委会审议的地方性法规草案18件，出台政府规章25件、规范性文件154件，修改和废止政府规章25件，全面完成“六五”普法工作。认真执行市人大及其常委会的决议、决定，自觉接受市人大及其常委会法律监督和工作监督，主动接受人民政协的民主监督和社会舆论监督，办理自治区和市级人大代表建议1440件、政协提案2418件，办结率100%；接受市人大常委会专题询问6件。

　　政府自身建设明显加强。坚决贯彻执行中央八项规定精神和国务院“约法三章”，深入开展创先争优、党的群众路线教育实践活动、“三严三实”专题教育和“两学一做”学习教育。纪检监察机关累计立案4622件、结案3985件、3861人受到党纪政纪处分、238人被移送司法机关处理，政府系统反腐倡廉工作取得新成效。主动公开政府信息30多万条。举办新闻发布活动620场（次）。持续开展市领导信访接待日、全市统一“公开大接访”等活动，畅通“市长热线”，共接听“市长公开电话”有效来电41.58万个，2015年人民网网友留言市长回复工作在副省级、地市级城市中位列第六。创新开展“两重两问”，有力促进政风转变。截至目前，举办31期《向人民承诺—电视问政》直播节目，314个问题全部整改落实。

　　五年来，我市司法、侨务、宗教、对台事务、审计、统计、人防、保密、水库移民、地震、供销、机关事务管理、地方志、档案、消防、海关、海事、检验检疫、税务、调查、通信、邮政、供电、测绘、气象、水文、参事、文史、哲学和社会科学等工作取得新进展，妇女儿童、残疾人、老龄等事业取得新进步。国防教育和后备力量建设深入开展，荣获“全国人民防空先进城市”。双拥共建工作取得新成绩，荣获“全国双拥模范城”六连冠，全国民族团结进步示范市创建工作成效显著。

　　五年来所取得的成就，充分展现了南宁的勃勃生机与活力，极大增强了全市各族人民的自信心和自豪感，必将激励我们在新的征程上继续前进，奋发图强，再创辉煌！

　　各位代表，五年成绩极其不易，这是党中央、国务院和自治区党委、政府以及市委坚强领导的结果，是全市各族人民团结奋斗、顽强拼搏的结果，是历届党委、人大、政府、政协班子打下良好基础的结果。在此，我代表市人民政府，向全市各族人民，向人大代表、政协委员，向各民主党派、工商联、无党派人士、各人民团体和各界人士，向驻邕人民解放军、武警部队官兵，向所有关心支持南宁建设发展的港澳台同胞、海外侨胞及海内外朋友们，表示衷心的感谢并致以崇高的敬意！

　　各位代表，回顾本届政府的工作历程，我们主动适应经济发展新常态，积累了在复杂环境中加快发展的宝贵经验；五年的不懈努力，创造了改革开放的累累硕果，取得了科学发展的辉煌成就。我们工作的主要体会是：一是必须坚持党的领导，坚守正确的政治方向，自觉维护市委总揽全局、协调各方的领导核心地位，切实增强政府执行力，真抓实干，克难攻坚，狠抓工作落实。二是必须坚持一张蓝图绘到底，把经过实践检验证明是正确的、有效的决策和举措坚持下来，与时俱进不断丰富和完善发展思路，科学确定发展目标。三是必须坚持以人民为中心的发展思想，把改善民生作为政府一切工作的出发点和落脚点，凝聚力量，共建共享。四是必须坚持全面深化改革，扩大开放，为经济和社会发展注入源源不断的动力和活力。五是必须坚持依法行政，全力推进民主法制建设，提高法治南宁水平，维护社会公平、正义。

　　各位代表，我们也清醒地看到，全市经济社会发展还面临一些突出的困难和问题：产业结构不优，工业短板明显，新旧动能转换尚未完成，发展后劲不足，对全区经济社会发展的支撑力、辐射力和先导带动作用不强；区域发展不够协调，大城市带大农村的现状没有改变，脱贫攻坚任务艰巨，还有不少民生诉求没有得到解决；政府自身建设有待进一步增强，等等。对于这些突出问题，我们必须高度重视，切实加以解决。

　　二、今后五年工作建议

　　今后五年，是全面建成小康社会的决胜期。我们将高举中国特色社会主义伟大旗帜，全面贯彻党的十八大和十八届三中、四中、五中全会精神，以马克思列宁主义、毛泽东思想、邓小平理论、“三个代表”重要思想、科学发展观为指导，深入贯彻习近平总书记系列重要讲话精神，紧紧围绕中央“五位一体”总体布局和“四个全面”战略布局以及广西“两个建成”战略目标，按照市第十二次党代会的决策部署，坚持发展第一要务，积极践行创新、协调、绿色、开放、共享的发展理念，以提高发展质量和效益为中心，大力实施产业转型升级、“南宁渠道”升级、绿城品质升级、深化改革升级、法治南宁升级、民生福祉升级“六大升级”工程，不断增创首府新优势，加快建设面向东盟开放合作的区域性国际城市、“一带一路”有机衔接的重要门户城市、对全区经济社会发展具有较强支撑带动作用的首府城市、具有浓郁壮乡特色和亚热带风情的生态宜居城市，勇当广西“两个建成”排头兵。

　　到2018年，全市地区生产总值比2010年翻一番（按可比价计算），一批重大产业和基础设施项目建成，“中国绿城”品质进一步彰显，城市形象大幅提升，以崭新面貌迎接自治区成立60周年。到2020年，地区生产总值突破5000亿元，人均GDP突破1万美元；城乡居民人均可支配收入比2010年翻一番；财政收入突破800亿元，占全区比重超过四分之一；全部工业增加值突破1600亿元，工业对经济增长的支撑作用进一步增强；第三产业增加值力争达到3000亿元，占全区比重接近30%，圆满完成“十三五”规划任务，打赢脱贫攻坚战，实现全面建成小康社会目标。到市第十四届人民政府任期结束时，基本建成“四个城市”，南宁成为广西改革创新的先锋、开放合作的先导、生态宜居的标杆、协调发展的典范、文明和谐的榜样，以优异成绩迎接建党100周年。

　　建设“四个城市”描绘了首府发展的美好蓝图，是自治区对南宁发展的战略定位，也是市委科学谋划南宁未来发展作出的重大抉择。“六大升级”工程是实现“四个城市”目标的具体举措，也是我市勇当广西“两个建成”排头兵的具体体现。我们必须把思想和行动统一到自治区党委、政府和市委的决策部署上来，开拓进取、万众一心、心无旁骛、埋头苦干，不断开创首府改革发展新局面。

　　围绕既定目标，提出以下工作建议：

　　（一）推动产业转型升级，在引领全区产业向中高端发展上实现新突破。

　　按照“二产补短板、三产强优势、一产显特色”的思路和要求，大力发展实体经济，推动产业迈向中高端。

　　补短板，打造区域性电子信息产业和先进制造业基地。落实《中国制造2025》，深入实施“工业强市”战略，加快形成3个千亿元产业、3个千亿元园区、5家百亿元企业。加快结构调整。做大做强电子信息、先进装备制造、生物医药三大重点产业。依托中国—东盟信息港南宁核心基地、富士康科技园等建设，大力发展中高端电子信息产品制造、软件和信息技术服务业，力争2020年电子信息产业产值实现2000亿元。以邕宁新兴产业园为主阵地，大力发展轨道交通装备、汽车及零部件等成套设备制造业和以铝精深加工为支撑的特色装备制造业；依托高新区发展智能制造、石墨烯等高端制造业，2020年先进装备制造产业产值达1200亿元以上。做强现代中药、生物制药、高端医疗器械等新兴产业，2020年生物医药产业产值达300亿元以上。同时，加快发展战略性新兴产业，2020年高技术产业产值力争达1500亿元。改造提升食品加工、化工、建材等传统优势产业。发展装配式建筑产业，促进建筑业转型升级。加快园区建设。实施园区经济倍增跨越计划，进一步拓展空间、完善功能，推进产城融合发展，构建“3+4+N”工业空间体系。高新区重点发展生物技术及健康产业、新一代电子信息、节能环保新技术及先进装备制造产业，经开区重点打造生物医药和临空产业，东盟经开区加快建设全区最大的食品加工产业基地，2020年三大国家级开发区工业总产值分别达1600亿元、1300亿元、700亿元。江南工业园区力争建成以富士康科技园为核心的产值千亿元电子信息专业园区，邕宁新兴产业园区建成以新能源汽车为主导产业的先进装备制造及临港产业专业园区，黎塘工业园区打造铁路关联产业园，六景工业园区打造循环经济特色明显的产业基地。同时，加快伊岭工业园区、良庆经开区、伶俐工业园区等县区工业园区建设。2020年全市工业园区规模以上工业增加值比2015年翻一番。培育强优企业。深入推进“抓大壮小扶微”工程，重点培育竞争力强、税收贡献大的企业，推动中小企业向专、精、特、新方向发展，形成百亿元企业为先导、十亿元企业为带动、亿元企业为支撑、中小微企业竞相发展的良好格局。

　　强优势，打造区域性现代服务业中心。做优做强金融服务、现代物流、电子商务和信息服务三大产业，统筹加快发展商务服务、科技服务、健康养老等新型服务业，优化提升商贸、会展等传统服务业，推动服务业发展提速、质量提高、结构提升。做优金融服务业。围绕打造区域性国际金融中心，深入实施“引金入邕”战略，争取中国进出口银行、平安银行、渤海银行等境内外金融机构在邕设立分支机构，吸引各类资本在我市设立股权投资基金和基金管理公司，健全金融机构组织体系。继续推动多层次资本市场体系建设，积极引入社会资本做大做强南宁金融资产交易中心、股权交易中心等平台。加大地方法人金融机构组建力度，推动组建市级农村商业银行，支持设立金融租赁公司、消费金融公司。坚持清理整顿和支持发展并重原则，引进一批运作规范的互联网金融企业。加快青秀区金湖金融中心、东盟商务区互联网金融产业基地等金融集聚区建设，提升金融业服务实体经济能力。2020年上市和新三板挂牌公司达50家，金融业增加值达750亿元左右。提升发展现代物流业。以构建物流大通道、建设城市配送网络、引进培育龙头企业、抓好物流集聚区建设为抓手，着力提升电商物流、农副产品物流、医药物流、钢铁物流、电子电器物流五大物流产业，重点推进南宁空港物流产业园、牛湾港综合物流园区等十大物流集聚区建设。2020年物流业增加值达470亿元。创新发展电子商务和信息服务业。推进国家电子商务示范市和国家信息消费示范市建设，实施“万企千店”电子商务推广工程、电子商务进农村工程等，加快中国—东盟电子商务产业园、中国—东盟（南宁）跨境电子商务产业园等园区建设，完善南宁跨境贸易电子商务综合服务平台，促进电子商务发展，2020年电商交易总额达3500亿元以上。实施“互联网+”战略，规划建设大数据产业园，发展云计算、大数据、物联网等新兴产业。同时，大力发展旅游业，依托环大明山旅游圈、邕江旅游带等特色旅游资源，打造一批精品旅游线路和特色旅游名县（镇、村），建设中国国际养生休闲旅游目的地。着力打造总部经济、楼宇经济、文化创意等一批集聚区，使之成为服务业发展的主战场和生力军。

　　显特色，加快发展现代特色农业。全面实施“10+3”特色农业产业提升行动，发展林药、林菌、林禽、林草等林业生态经济，构建现代特色农业产业体系。继续实施耕地质量保护和提升行动，确保粮食总产量稳定在222万吨以上。加快建设现代特色农业示范区，到2017年建成自治区级示范区10个和自治区认定的县（区）级示范区20个、乡镇级示范区30个。坚持产村互动、农旅融合，大力发展都市休闲农业，2020年我市成为广西都市休闲农业发展的样板。持续实施农业品牌化战略，2020年“三品一标”认证农产品超过250个，以淘宝、京东等第三方特产馆平台建设为载体，培育农产品网络销售品牌。加快农业科技成果转化应用和技术推广，深入实施种子工程和水产畜禽良种工程，高标准建设一批良种繁育基地，2020年主要农作物良种覆盖率达96%以上、畜禽良种对生产贡献率达80%以上、科技进步对农业增长贡献率达62%。完善农业新型社会化服务体系，引导土地向新型农业经营主体流转，2020年全市专业合作社达5000个、家庭农场达1000家。逐步完善农业保险制度，2020年全市主要农产品政策性农业保险参保率达50%。加快以水利为重点的农业基础设施建设，夯实农业发展基础支撑。积极稳妥推进农村各项改革，2018年底基本完成农村土地确权工作。加强农产品质量安全监管，确保“舌尖上的安全”。

　　优服务，提升投资有效性和企业竞争力。突出抓好产业、民生、基础设施三大类投资，着力提高技改投资比重，力争固定资产投资年均增长11%。积极拓宽项目融资渠道，发挥好产业发展基金、城市发展基金的引导作用，探索设立各类引导基金；在重点领域推广PPP模式，鼓励社会资本以多种形式参与公共基础设施项目的投资、建设和运营；引导企业利用发行企业债、项目收益债券、短期融资券和中期票据等解决资金瓶颈问题，增加民间投资和外来投资比重。推进三大国家级开发区行政审批局二次改革，推动市行政审批局规范运行，提高审批效率。坚持实施市领导联系重大项目服务重点企业制度，按照定人员、定职责、定时间、定进度的“四定”要求，完善重大项目服务推进机制；大力实施降低实体经济企业成本行动，为企业减负，为市场松绑，打造“亲”“清”新型政商关系。发展普惠金融，加大金融对中小微企业、“三农”等的扶持力度。大力实施产业工人技能培训，年均培训万人以上。

　　（二）推动“南宁渠道”升级，在引领全区全方位开放合作上迈出新步伐。

　　按照自治区“四维支撑、四沿联动”开放合作布局要求，突出平台支撑、交通支撑、产业支撑，努力构建全方位、宽领域、多层次的开放发展新格局。

　　突出平台支撑，拓展开放合作渠道。积极参与“一带一路”建设，扎实推进与泛北部湾区域和澜沧江—湄公河合作等次区域的合作，服务好中国—东盟博览会和商务与投资峰会升级版建设。落实“双核驱动”战略，推动中国（北部湾）自贸试验区创建，深入推进南北钦防同城化，促进北部湾经济区升级发展；积极落实粤桂共同行动计划，推进CEPA示范城市建设。扎实推进市级对外开放合作平台载体建设，加速中国—东盟信息港南宁核心基地规划和建设，加快搭建基础设施、信息共享、技术合作、经贸服务、人文交流五大平台，打造21世纪“海上丝绸之路”的重要信息枢纽。充分利用南宁综合保税区，重点发展电子信息、保税物流加工、跨境电商等产业，建成面向东盟的进出口重要渠道。加快空港经济区规划建设，加快发展空港物流、航空维修制造、临空高新技术等产业，打造面向东盟的区域性国际航空物流中心和现代化临空产业基地。

　　突出交通支撑，夯实互联互通基础。打造区域性国际综合交通枢纽中心，完善现代铁路网络，配合自治区重点推进泛亚铁路中东线、贵阳至南宁客运专线、湘桂铁路南宁至凭祥段扩能改造、南宁至呼和浩特高铁北上第二通道等一批铁路项目建设；加快建设南宁五象站和北站，完善南站，改造南宁站，提升东站综合交通枢纽功能。完善出省、出海、通口岸高速公路主通道网络，加快柳南高速公路改扩建、柳州经合山至南宁、吴圩机场经大塘至浦北、贵港经隆安至硕龙等高速公路建设；规划建设五象客运站，建成东站综合客运枢纽，提升玉洞等货运站场。推进吴圩机场军民分离，按F类机型建设第二跑道，规划建设T3航站楼，完善机场第二高速配套设施；加密空中国际航线，实现东盟国家主要城市航线全覆盖，争取吴圩机场72小时过境免签，建成面向东盟的门户枢纽机场；加快建设伶俐通用机场。建成邕宁水利枢纽等，完成西津水利枢纽二线船闸工程，完善港口集疏运系统，推动铁路、公路、水路联运，2020年我市港口吞吐能力达2000万吨、千吨级以上泊位43个、集装箱吞吐能力5万标箱。

　　突出产业支撑，强化对外经贸合作。融入高铁经济带建设，加强与珠三角、长三角和西南中南地区在对外贸易、资源开发、交通物流、文化旅游等领域的合作。加深与东盟国家在农业、机械制造、食品加工等产业领域的合作，重点抓好中国（南宁）—文莱农业产业园、中国—东盟现代农业示范园等园区建设，建成一批产业合作示范基地。建设中国服务外包示范城市，大力发展软件和信息技术、金融、生物医药、文化创意设计等领域服务外包，力争服务外包合同执行额年均增长15%以上。扩大先进技术、关键零部件和重要资源性产品等的进出口，培育出口生产基地和出口名牌，带动自主品牌出口。鼓励有条件的企业在境外投资建厂，支持有条件的企业通过境外上市、并购等方式参与国际分工。

　　实施精准招商，提升引资成效。深入实施招商引资“三年行动计划”，瞄准世界、中国、民营500强企业和行业领军企业，面向粤港澳台、欧美日韩、新兴经济体、“一带一路”沿线国家，精准招高招新招强。围绕打造产业链，抓好重点企业、重点产业、重点区域精准有效招商，实现招商引资扩量提质。创新招商引资方式，大力开展国企混合所有制改革招商、平台招商、以商引商等，积极探索产业园区共建、园区整体开发招商、委托招商、境外融资、“互联网+招商”等招商方式，科学策划包装项目，加快建立招商项目库、客商信息库、政策库，做好重大项目储备。力争全市区外境内到位资金、全口径实际利用外资、战略性新兴产业到位资金年均增长10%。

　　（三）推动绿城品质升级，在引领全区生态宜居城市建设上打造新亮点。

　　落实中央、自治区城市工作会议的部署要求，坚持治水、建城、为民主线，注重水绿、产城、新旧、建管、“里”“面”“五个融合”，转变城市发展方式，打响面向东盟品牌、提升生态宜居品牌、拓展民族文化品牌。

　　强化城市规划引领。突出规划的全局性、科学性、前瞻性和权威性，启动新一轮城市总体规划修编，构建以城市总体规划为统领、以控制性详细规划和城市设计为抓手、专项规划配套、各层次规划紧密衔接的城市规划体系，加快推进城市总体规划和土地利用总体规划“两图合一”，探索建立“多规合一”体系。树立规划即法的理念，强化规划执行。加强城市设计，对重点区域空间形态、公共空间、景观风貌等进行控制和引导，保护和传承旧城区整体历史环境和风貌，注重保留和突出南宁特色的文化基因，打造具有壮乡民族特色和东盟文化融合共生的城市风貌。

　　大力推进城市建设。统筹城市地上和地下建设，有效防范和化解大雨内涝、交通拥堵、马路拉链、空中蛛网等城市问题，筑牢城市运行保障线。推动城市水绿融合。依法保护利用邕江、青秀山和五象岭“一江两山”的宝贵资源，打造“中国绿城”升级版。把“治水”放到更重要的位置，抓好邕江综合治理，整体改造城市内河水系，推进各类滨水绿地公园建设，完善生态、商贸、旅游等功能，统一规划邕江两岸建筑风格，打造融“江、滩、堤、路、城”于一体的滨江景观。推进国家海绵城市试点建设，落实三年实施计划，加快城市东西向快速路海绵化改造等130多项海绵项目建设，2020年建成区20%以上的面积达到就地消纳和利用70%降雨的目标。大力推进全国水生态文明试点城市建设，全面完成市区易涝点整治，全面开展生活污水直排口整治和建成区黑臭水体治理，加快城市污水管网和建成区雨污分流改造，实施城市供水专项规划，加快城市取水口上移、应急水源及供水管网建设。进一步增强建筑节能管理，新建民用建筑全面推行绿色建筑标准。深化国家森林城市和国家生态园林城市建设，办好第十二届中国国际园林博览会，打造一批绿化重点区域和精品线路，推进智慧林园和公园“一园一品”建设，提升绿城品质。优化城市交通网络。提高路网密度，2020年建成区平均路网密度接近8公里/平方公里，道路面积率不低于15%。加快推进高改快工程、东西向和南北向快速路建设，继续推进跨江桥梁、立交桥等基础设施建设。实施断头路建设攻坚战，2018年全面打通市区重要交通节点断头路。统筹规划和建设停车场、停车泊位，有效缓解停车供需矛盾。建成轨道交通1至4号线，力争建成2号线东延长线和5号线，完成轨道交通第三轮建设规划。创建“国家公交都市”，加快快速公交试点工程、公交专用道、常规公交场站等建设，形成轨道交通、快速公交、常规公交、慢行交通等无缝衔接的公交体系，2020年公共交通分担率达30%以上。提升城市公共服务水平。加快推进市图书馆等16个迎接自治区成立60周年大庆重点项目。实施地下综合管廊试点城市五年项目滚动规划，加快建设凤岭北路综合管廊等40多项管廊项目，建成综合管廊110公里以上。加快推进棚户区改造和“老南宁·三街两巷”等特色街区建设。到2020年，实现棚户区改造开工56692户，基本完成建成区棚户区改造任务。改善西乡塘等西部片区的基础设施。着力完善高密度人口聚集区的公共服务配套设施，加强广场、绿地等公共空间建设，2018年基本完成社区居家养老服务中心、幼儿园、文化室、卫生服务中心和体育场所等“五个一”公共服务工程建设，2020年社区“15分钟生活圈”基本覆盖建成区。

　　提升城市治理水平。着力提升城市治理法治化、精细化、智慧化水平。完善城市管理法律法规和标准体系，推进全国综合行政执法体制改革试点城市建设，逐步实现住房城乡建设领域的全部行政处罚权和环境保护等部分行政处罚权及有关行政强制权的相对集中行使，建立城市管理联合执法平台，提高执法水平。适应群众个性化、差异化需求，建立城市建筑物、构筑物、道路、绿化及各类公共设施等城市管理单元的管理标准，明确管理制度和质量指标。推进城市治理网格化，实行网格长负责制。应用新一代信息技术，整合优化和完善现有信息系统，让“智慧南宁”点亮城市生活。把“美丽南宁·整洁畅通有序大行动”工作经验固化为城市治理长效机制。加强城市公共管理，保障供水、供电、供气、交通救援线路等城市生命线系统畅通，加强建筑工程质量安全控管，切实做好消防、抗震、防洪、气象、地质、水文、人防等城市安全工作。完善应急响应机制和应急预案，提高应急处置能力。依法打击违法用地、违法建设行为。加强渣土运输市场监管。落实居住证制度，扩大基本公共服务覆盖人群。发挥市民主体作用，鼓励和支持市民参与城市管理，实现城市共治共管、共建共享。

　　加速五象新区建设。围绕“再造一个新南宁”的战略目标，以加快创建国家级新区为抓手，着力拓空间、强功能、兴产业、提品质。推动新区与空港经济区及周边区域联动融合发展，2020年核心区全面建成，新区基础设施和公共服务设施基本完善，青山大桥等一批跨江桥梁建成，次干路网全面完善、片区路网基本建成，完成一批垃圾处置、污水管网、公交场站等市政配套项目建设，广西国际壮医医院、十四中五象校区等一批知名医院和中小学校建成使用。自治区重大公益性项目片区、蟠龙片区、玉洞片区、龙岗片区、文旅组团、园博园组团和那马组团等各大片区组团开发建设全面推进。“两基地一中心一商圈”建设加快，总部基地金融街、中国—东盟国际物流基地、邕宁新兴产业园等产业园区基本成型，初步形成现代产业体系，实现产城融合发展。率先推行城市设计，发展新型建造方式，推广街区制，推进地下空间综合利用，提升新区绿色宜居、文化绽放、产业集聚的品质魅力。

　　推动县域转型发展。落实主体功能区规划，推进区域差异化发展。加快新型城镇化进程，坚持扩容提质与凸显特色并重，推动县城向城市转型，培育一批特色小城镇。统筹推进现代产业向县域协同分工，做大做强县域支柱产业、特色产业，壮大县域经济规模，横县做强电力、化工、造纸和机械制造等产业，建设广西重要工业基地和广西现代特色农业强县，打造滨江宜居中等城市；宾阳加快发展铁路关联产业，做优食品、林木加工、新型建材及轻纺制品等产业，着力打造民营经济强县、构建现代化商贸名城、建设宜居宜业中等城市；上林着力发展特色旅游，打造全国全域旅游示范县、广西特色旅游名县、全国生态文明建设示范县，彰显南宁“后花园”风采；马山注重保护生态与脱贫攻坚有机结合，重点发展生态休闲旅游与养生运动产业，创建广西特色旅游名县，打造全国生态文明先行示范区；隆安着力培育生物医药等新兴产业，发展现代特色农业，建设广西扶贫生态移民试点县。统筹推进交通设施向县域便捷联通，规划建设连接横县、宾阳、上林、马山、隆安的都市圈高速公路环线，积极推动中心城区至外环高速公路等快速通道建设；大力推进新江至扶绥一级公路、国道G322五塘收费站至宾阳（黎塘）二级路改一级路、宾阳至上林二级路改一级路、张村至六景公路等项目建设，畅通县域交通网络。统筹推进公共服务向县域精准延伸，加快光网南宁、无线南宁、移动互联网、下一代广播电视网建设，实现互联网城域出口、用户接入能力提升，2020年中心城区及县城免费无线网络全覆盖；加快电网、油气管网等建设，全面完成“县县通”天然气工程；推进各县的主要支流河段防洪堤建设，争取县城防洪标准达到20年一遇。

　　加强环境保护治理。推进绿色发展，建设天蓝地绿水清的生态环境，创建国家环保模范城市。实行最严格的环境保护制度，加强环境监测和环境执法，深入实施大气、水、土壤污染防治三大行动计划，重点推进扬尘污染治理专项行动，开展燃煤、机动车尾气治理，严控污染物新增排放量；实施最严格的水资源管理制度，推进水污染防治、水生态保护，强化对邕江、大王滩水库等市级饮用水源地及449个千人以上农村饮用水源地的保护管理；加强土壤环境保护及修复、重金属污染防治和风险管控，加大农业面源污染防治力度。建立覆盖全市的餐厨垃圾收运处理系统，推进生活垃圾分类。控制城市开发强度，划定水体保护线、绿地系统线、基础设施建设控制线、历史文化保护线、永久基本农田和生态保护红线。大力推进土地节约集约利用，到2020年我市节约集约模范县（区）达6个以上。强化对生态敏感区域的保护和管理，推进城市修补和生态修复。严格环境准入，淘汰落后产能，重点建设东盟经开区国家循环化改造示范试点园区，推进高新区创建国家低碳工业试点。深入开展“美丽南宁”乡村建设，着力改善农村人居环境。

　　（四）推动深化改革升级，在引领全区改革创新发展上推出新经验。

　　突出问题导向，聚焦重点领域，推出一批重大改革举措，推动全面创新，进一步解放和发展生产力。

　　推进供给侧结构性改革。按照优化存量、引导增量、有效减量的要求，有力、有度、有效落实好“三去一降一补”重点任务，促进经济社会持续健康发展。继续积极稳妥化解过剩的玻璃、木薯淀粉酒精等产能，推动资源优化配置，促进制糖、造纸、建材、淀粉等行业循环经济发展。认真做好商业、办公用房去库存工作，调整商品房供应结构，加强市场监管，促进房地产市场健康发展。全面落实降低企业税费负担、融资成本、制度性交易成本、能源成本、物流成本等措施，建立和完善降低实体经济企业成本的长效机制。补上脱贫攻坚及现代基础设施、教育文化卫生供给等公共服务短板，努力增加有效供给。推进简政放权、放管结合、优化服务工作，清理规范涉及市、县级行政审批的中介服务事项，加强事中事后监管。加大政府部门权责清单制度推行力度，全面推行乡镇政府（街道办事处）权责清单。深化商事制度改革。

　　深化重点领域改革。深入推进财税改革。改进年度预算控制方式，加强预算执行管理，健全预算绩效评价管理机制，规范政府性债务管理，进一步理顺收入划分、事权和支出责任，完善转移支付制度，落实省直管县财政管理制度，多措并举防范基层财政风险。加快落实增值税改革，推进消费税、资源税等改革，全面落实税费优惠政策。推动金融改革创新。加快推进沿边金融综合改革试验区建设，推动区域性跨境人民币业务平台（南宁）与“一带一路”沿线国家和地区支付清算系统的合作，支持银行业金融机构和支付机构为东盟国家提供跨境支付服务。提升南宁跨境金融信息服务基地功能。争取国家丝路基金、中国—东盟海上合作基金项目落地。鼓励新兴金融业态规范有序发展，创新金融产品，发展产业金融、科技金融等。深化农村金融改革，加大农业担保投入力度，探索开展农村产权抵押贷款试点等。深化国资国企改革。分类推进国企改革，完善现代企业制度，进一步健全法人治理结构。推动以管资本为主的国有资产监管体制建设，完善国企分类考核评价体系和企业薪酬分配制度。优化国有经济布局，提高国有资本配置效率，重点向战略性新兴产业、现代服务业、基础设施等关键领域集中。深化教育领域综合改革。推进教育管办评分离、人才培养、考试招生和学校办学体制改革。深化医药卫生体制改革。进一步推动医疗、医保、医药三医联动。同时，稳步推进事业单位分类改革，加快推进户籍制度、供销合作社综合改革等重点改革，让市民更多分享改革红利。

　　大力推动创业创新。深入实施创新驱动战略，创建国家自主创新示范区，加快建设以企业为主体的技术创新体系。健全技术创新、科技服务创新等政策体系，新增一批国家和自治区级重点实验室、工程技术研究中心、特色园区、技术转移中心、国际科技合作基地、产业技术创新战略联盟和科技服务机构。改革和创新科研经费使用和管理方式。尽快建成集展示、交易、交流、合作、共享于一体的创新成果市场。创建国家知识产权示范市，推进建设“中国—东盟知识产权国际交流合作中心”。推动大众创业、万众创新，加快发展“互联网+”创业网络体系和众创、众包、众扶、众筹。紧扣产业链和创新链，创新人才政策，完善人才服务机制，加强海外人才合作，大力引进培养高层次人才。加快南宁·中关村双创示范基地建设，植入中关村创新基因和机制，集聚一流的人才、技术等创新创业资源和要素，打造创新创业服务平台，建设战略性新兴产业集聚发展的科技中心、示范中心和服务中心。2020年，全社会研究与试验发展经费支出占地区生产总值比例达2.2%左右，每万人发明专利拥有量达8件以上，全市科技进步对经济增长的贡献率达60%。

　　（五）推动法治南宁升级，在引领全区法治政府建设上打开新局面。

　　贯彻落实《法治政府建设实施纲要（2015—2020年）》，建立并实施法治政府建设指标体系，把政府工作全面纳入法治轨道。

　　改进政府立法。突出立法引领和推动作用，坚持立法和改革决策、经济社会发展相衔接。推进立法精细化，完善立法立项起草、论证、协调、审议机制，优化委托第三方起草等多元化立法起草工作机制，完善重大立法问题向市委报告制度。坚持科学民主立法，完善立法机关主导、有关部门参加和人大代表、政协委员、专家学者共同参与的立法工作机制；完善社会各方有序参与政府立法的途径和方式，健全公众意见采纳情况反馈机制，广泛凝聚社会共识。健全政府立法后评价机制。扎实推进规章修改、废止、解释、清理等工作。严格规范性文件制定，健全并落实规范性文件“三统一”制度，强化规范性文件事前合法性审查和事后备案审查。实行政府规章、规范性文件目录和文本动态化、信息化管理。严格落实《南宁市重大行政决策程序规定》，有效发挥政府法律顾问作用，提高专家论证和风险评估质量。

　　公正文明执法。严格实行行政执法人员资格管理制度。建设行政执法程序标准化、标准实施流程化、流程控制信息化、监察管理全程化的行政执法暨电子监察系统。严格规范行政许可、处罚、强制、征收、收费、检查等执法行为，做到公正严明。建立健全违法线索发现、违法行为查处、行政执法监督相衔接的工作机制。全面建立健全行政裁量权基准制度。提高行政执法行为规范化水平，建立对立案、调查取证、告知、听证、集体讨论决定等行政执法活动全过程记录制度。严格执行重大行政执法决定法制审核制度。推进文明执法，积极推行全程说理式执法、行政监管劝勉等柔性执法方式。加大对重点领域违法违规行为和阻挠执法行为的打击力度。建立健全违法失信惩戒机制，推动形成诚实守信、尊法守法的良好社会风尚。

　　强化行政权力制约监督。自觉接受市人大及其常委会法律监督和工作监督，主动接受人民政协的民主监督，完善政府重大事项决定前向人大报告制度，认真办理人大议案、代表建议和政协提案。支持司法机关依法监督，推行行政机关负责人出庭应诉制度。建立落实人民法院、人民检察院司法建议制度，尊重并执行人民法院的生效判决、裁定，认真对待人民法院的司法建议，配合检察机关对在履行职责中发现的行政违法行为进行监督。强化政府内部监督，及时纠正违法或者不当的行政行为。推进舆论监督和社会监督，提升应对网络舆情能力，建立和完善群众举报投诉制度、行政执法监督员制度，充分发挥电视问政、政风行风热线等监督功能。加强专门监督，对直接分配财政资金、直接接触工程建设、直接面向群众服务等重点领域和关键环节实现流程规范控制全覆盖。全面推进政务公开。全面推行办事公开制度，完善首问负责、服务承诺、一次性告知、限时办结等制度。建成全市统一的互联网信息服务平台，实现市县乡三级政府网络互联。

　　营造崇尚法治环境。加强法制机构和队伍建设，建立领导干部任职前依法行政情况考察和法律知识考试制度，完善法治培训和学法制度。建立领导班子和领导干部述法制度。深入开展“七五”普法工作，积极推动法律“六进”，建设一批法治文化阵地，充分运用新媒体开展法治宣传，打造“互联网+法治宣传+法律服务”新平台。深入开展青少年法治宣传教育，统筹强化学校、家庭和社会三位一体的教育格局。完善人民调解、行政调解、司法调解联动工作体系，建立依法维权和调处化解矛盾纠纷综合机制。推进城乡一体化的公共法律服务体系建设，建立完善市县乡村四级法律服务平台。

　　（六）推动民生福祉升级，在引领全区基本公共服务供给上取得新成效。

　　坚持以人民为中心的发展思想，努力增进民生福祉，让发展成果最大程度实现人民共享。

　　坚决打赢脱贫攻坚战。深入实施“七个一批”“七大工程”，落实2017—2019脱贫摘帽滚动计划，加大资金投入，发挥群众脱贫主体作用，确保如期完成脱贫任务。突出产业扶贫，按照“合作社（龙头企业）+经济能人”等模式，大力发展特色种养业、乡村旅游业、农村电商。推进转移就业扶贫，加快完善就业创业培训、服务、维权机制，提高建档立卡贫困户劳动力就业脱贫能力。实施易地搬迁扶贫，按照政府主导、群众自愿的原则，有计划地实施易地扶贫搬迁10.93万人，确保搬得出、稳得住、可发展、能致富。推进生态扶贫，积极推广生态保护、惠民脱贫的“弄拉模式”，鼓励生态保护性开发投资，惠及贫困群众。深化教育扶贫，落实建档立卡贫困户子女的15年免费教育和优先享受教育资助政策。推进医疗保险和救助扶贫，扶持建档立卡贫困人口参加新型农村合作医疗，规范开展医疗救助和临时救助工作。推进政策性保障兜底扶贫，加快农村低保制度与扶贫开发政策、对象、保障标准、补助水平、管理、工作机制、信息数据、社会力量的“八个衔接”。落实市统筹、县（区）负主责，部门衔接联动、倾斜推动，单位定点帮扶，干部到户帮扶等脱贫攻坚责任机制。建立到村到户帮扶工作机制，深入开展“一帮一联”。完善“三承诺”“双确认”“双认定”第三方评估机制，提高帮扶成效。健全扶贫投入增长、监管机制和社会扶贫机制。建立贫困户帮扶信息“一户一册一卡”，完善扶贫信息管理系统。健全科学合理的脱贫验收评估机制和动态统计监测机制。完善脱贫攻坚督查、考核、奖惩制度，确保脱贫攻坚措施落实到位。

　　着力提高群众生活质量。实施城乡居民收入倍增计划，多渠道促进城乡居民增收。落实中央扩大中等收入群体、深化收入分配制度改革的政策措施，形成公正合理的收入分配格局。建设充分就业城市，完善就业服务体系，推进高校毕业生、农民工等重点群体就业，落实创业帮扶政策，推进创业带动就业。完善社会救助体系，落实最低生活保障标准动态调整机制，提升特困供养服务水平，落实重特大疾病医疗救助、临时救助等制度。构建以公共租赁住房为主的保障性住房供应体系，解决中低收入群体的住房保障问题。

　　大力推进教育现代化。促进教育公平协调发展，鼓励发展多元普惠幼儿园，推进县（区）义务教育均衡发展，开展义务教育学区制改革和集团化办学，推动普通高中多样化、特色化发展，推进职业教育产教融合发展，在全区率先普及十五年教育。优化城乡学校布局，推进“全面改薄”“双千计划”项目建设，改善办学条件，缩小城乡、区域、校际教育发展差距，统筹做好特殊教育、农村留守儿童教育、进城务工人员随迁子女教育等工作。建立与驻邕高校良性互动、融合发展机制，提升南宁学院、南宁职业技术学院等市属高校的办学水平，力争南宁学院升格为南宁大学。加快南宁教育园区建设，打造产学研一体的高级人才培养基地和科研文化交流中心，推进中高职一体化人才培养。推动信息技术与教育教学深度融合，实现教育信息现代化上台阶。优化教师队伍结构。鼓励和引导区内外学校、社会力量、民间资本按照规划布局和办学标准参与我市各类教育建设，争取与更多的名校、名师合作。2020年学前三年毛入园率达97%，九年义务教育巩固率达96%，高中阶段毛入学率达95%。

　　建设健康南宁。贯彻落实“健康中国2030”规划纲要，着力提高人民健康水平。普及健康生活，大幅提升城乡体育设施供给，构筑城市社区10分钟健身圈，2020年人均体育场地面积达1.8平方米，促进居民身体素质提升。优化健康服务，全面落实政府办医责任，推进公立医院综合改革，加快建立分级诊疗制度，打造医疗联合体；加强基层卫生服务能力建设，优化医疗卫生资源布局；扶持中医药、壮瑶等民族医药事业发展，吸引社会资本举办高端医疗机构和特色专科医疗机构，鼓励设立合作医疗机构。完善健康保障，推进全面两孩政策实施，促进人口长期均衡发展；实施全民参保计划，扩大社会保险覆盖范围，完善各项社会保险制度，推进机关事业单位养老保险制度改革，确保2020年全市基本养老保险、基本医疗保险参保率分别达90%、95%以上。建设健康环境，巩固提升国家卫生城市创建成果，加大环境卫生综合整治力度，抓好疾病防控、除“四害”等工作。发展健康产业，加快体育产业发展，促进体育消费，倡导健康生活方式；大力发展养老产业，促进医养结合，2020年全市千名老人拥有养老床位数达40张以上。

　　提升文化软实力。深化全国文明城市创建，大力培育和践行社会主义核心价值观，加强公民道德素质教育，推进社会诚信体系建设，提高社会文明程度。提高公共文化服务水平，完成广西文化艺术中心、南宁顶蛳山遗址博物馆等一批文化事业项目建设，引导公共文化资源向基层延伸，基本建成市县乡村四级公共文化设施网络体系；扶持文艺创作，打造一批体现地方民族特色的艺术精品。深化对外文化传播交流合作，精心组织开展中国—东盟民族文化论坛、中国—东盟（南宁）戏剧周、“文化走亲”东盟行等系列活动。扶持文化产业发展，打造文化产业品牌，建设具有重要影响力的区域文化中心。积极创建国家历史文化名城，加强历史文化街区、历史建筑、古镇、古村落等历史文化遗产以及非物质文化遗产的保护和开发，将文化资源转化为发展优势。加强哲学、社会科学和地方史志研究。

　　加强和创新社会治理。深入推进平安南宁建设，积极推进“智慧警务”，扎实做好反恐防暴工作，坚决打击“两抢一盗”、黄赌毒、传销、网络诈骗等违法犯罪行为，加快构建全方位、立体化的社会治安防控体系。全面铺开社区网格化治理模式，增强社区服务功能。鼓励支持社会组织参与社会治理和公共服务。健全完善矛盾纠纷调解机制，及时将矛盾纠纷解决在基层、消除在萌芽状态。加强和改进信访工作，解决好群众合理合法的利益诉求，依法处置信访活动中的违法犯罪行为，维护正常信访秩序。发挥人民网留言、市长公开电话、市长信箱的作用，及时回应社会关切。推动民族团结进步创建活动。严格实行安全生产党政同责、一岗双责、失职追责，完善监管体制，有效预防和坚决遏制重特大事故发生。全面加强食品药品监管能力和应急处置能力建设，创建国家食品安全城市。推进南宁气象现代化建设。推进社区矫正规范化建设，加强流动人口和特殊人群管理。加强国防动员和后备力量建设，增强全民国防意识，扎实做好双拥工作，推动军民融合深度发展，支持军队改革。

　　坚持为民办实事。坚持每年承办并完成自治区级和市本级为民办实事工程，着力解决出行难、停车难、空气质量优良保持难等一批关乎老百姓切身利益的问题，让发展成果更多惠及百姓。

　　三、加强政府自身建设

　　始终牢记人民公仆意识，主动适应形势发展新要求，转变工作作风，提高政府公信力和执行力，以良好形象取信于民。

　　（一）从严治政，加强廉政建设。

　　坚持把纪律和规矩挺在前面，严格落实党风廉政建设责任制和领导干部廉洁自律各项规定。持之以恒贯彻落实中央八项规定精神，严厉查处违纪违法行为，坚决惩处重点领域腐败和整肃庸政懒政怠政。健全权力运行监管机制，严格公共资金、国有资产、国有资源和领导干部履行经济责任情况的审计监督，规范公共资源交易管理服务平台建设，实现交易服务全过程网上留痕、交易监管全过程网上监控，减少权力寻租空间，从源头上预防腐败。严肃财经纪律，加大对重点领域、重点资金、重点部门的审计力度，强化领导干部经济责任审计。严控“三公”经费支出，将有限财力更多用于补齐民生短板。

　　（二）执政为民，提升服务水平。

　　推进行政机构、职能、权限、程序、责任法定化，依法规范行政机构设置和部门职责。深入推进政企分开、政资分开、政事分开、政府与市场中介组织分离，实现人员、机构、财务、资产、职责“五分开”。推进政务服务规范化建设，建立健全市县乡三级政务服务中心标准化体系。支持行业协会商会、志愿服务等社会组织发展，引导基层群众自治组织建立健全自治制度，逐步实现“政社互动”全覆盖。贯彻落实国家“互联网+政务服务”战略，加快建设一站式社会服务管理平台。坚持问政于民，深入基层一线调查研究，使政府工作、政策措施更加符合民意。

　　（三）狠抓落实，提高执行能力。

　　深入践行党的群众路线，落实“三严三实”要求，开展“两学一做”学习教育，促进干部队伍作风转变。健全科学合理的绩效考评体系和考评机制，强化效能监督，深化“两重两问”工作。倡导求真务实作风，健全激励机制和容错纠错机制，营造干事创业的良好环境。推进学习型机关建设，增强创新意识，提高干部队伍履职能力，推动工作落实。

　　各位代表，今年以来，我们认真贯彻落实党中央、国务院和自治区党委、政府以及市委的各项决策部署，紧紧围绕稳增长各项目标任务，采取有力措施积极应对各种风险挑战，经济社会保持良好发展态势。上半年，全市地区生产总值1615.76亿元、增长6.9%，农林牧渔业总产值205.8亿元、增长3.5%，规模以上工业增加值470.91亿元、增长4.8%，固定资产投资1624.99亿元、增长10.53%，社会消费品零售总额904.18亿元、增长10.2%，财政收入320.78亿元、增长10.68%，二氧化硫、化学需氧量、氨氮和氮氧化物减排、节能降耗均按自治区政府下达的任务全面实施，脱贫攻坚工作扎实推进，社会各项事业全面发展，民生持续改善。今年最后两个多月，我们将着力推动产业转型升级，着力服务项目和企业，着力提升生态宜居水平，着力推进改革创新，着力扩大开放合作，着力保障改善民生，继续抓好稳投资、稳工业、稳农业、稳消费、稳进出口、稳财政工作，坚定信心、狠抓落实，确保顺利完成年度各项目标任务，努力实现“十三五”良好开局。

　　各位代表！宏伟蓝图催人奋进，新的征程任重道远。让我们紧密团结在以习近平同志为总书记的党中央周围，高举中国特色社会主义伟大旗帜，在自治区党委、政府和市委的正确领导下，凝聚全市人民的智慧和力量，不忘初心、继续前进，奋发有为、攻坚克难，为加快建设“四个城市”、勇当广西“两个建成”排头兵而努力奋斗！
